# Ru Catalysts Supported on Bamboo-like N-Doped Carbon Nanotubes: Activity and Stability in Oxidizing and Reducing Environment

**DOI:** 10.3390/ma16041465

**Published:** 2023-02-09

**Authors:** Arina Korobova, Nikolay Gromov, Tatiana Medvedeva, Alexander Lisitsyn, Lidiya Kibis, Olga Stonkus, Vladimir Sobolev, Olga Podyacheva

**Affiliations:** Boreskov Institute of Catalysis SB RAS, Lavrentiev Av. 5, 630090 Novosibirsk, Russia

**Keywords:** carbon nanotubes, bamboo-like, nitrogen doping, ruthenium, stability, CWAO, phenol, formic acid, hydrogen

## Abstract

The catalysts with platinum-group metals on nanostructured carbons have been a very active field of research, but the studies were mainly limited to Pt and Pd. Here, Ru catalysts based on nitrogen-doped carbon nanotubes (N-CNTs) have been prepared and thoroughly characterized; Ru loading was kept constant (3 wt.%), while the degree of N-doping was varied (from 0 to 4.8 at.%) to evaluate its influence on the state of supported metal. Using the N-CNTs afforded ultrafine Ru particles (<2 nm) and allowed a portion of Ru to be stabilized in an atomic state. The presence of Ru single atoms in Ru/N-CNTs expectedly increased catalytic activity and selectivity in the formic acid decomposition (FAD) but had no effect in catalytic wet air oxidation (CWAO) of phenol, thus arguing against a key role of single-atom catalysis in the latter case. A remarkable difference between these two reactions was also found in regard to catalyst stability. In the course of FAD, no changes in the support or supported species or reaction rate were observed even at a high temperature (150 °C). In CWAO, although 100% conversions were still achievable in repeated runs, the oxidizing environment caused partial destruction of N-CNTs and progressive deactivation of the Ru surface by carbonaceous deposits. These findings add important new knowledge about the properties and applicability of Ru@C nanosystems.

## 1. Introduction

Carbon nanomaterials as catalyst supports have attracted high interest for a long time since they allow the controlled synthesis of supported active components [1,2]. At the same time, one of the most important aspects is the stability of carbon-supported catalysts, which will determine the area of possible application of these materials.

Among Pt-group metals, Pt- and Pd-based catalysts commonly attract particular attention owing to their wide application in various industrial processes, although the use of cheaper metals is highly desirable. Ruthenium, whose cost is 2–5 times lower compared to Pt and Pd, can be considered a worthy alternative in some cases. For example, Ru/C catalysts were found to be highly active in the decomposition [3] and hydrogenation [4] reactions. Indeed, promising results were obtained in the catalytic transfer hydrogenation reactions by utilizing propanol as a source of hydrogen [5,6]. Formic acid (FA) can also be used as a source of hydrogen; for example, levulinic acid was successfully reduced to γ-valerolactone at 150 °C [7] or C6-sugar sources to γ-valerolactone at 180 °C [8] on 5%Ru/C. Indeed, earlier we showed that the gas-phase FA decomposition (FAD) proceeds on the 1%Ru catalyst deposited on N-doped carbon nanofibers (N-CNFs) with the selectivity to hydrogen up to 91.5% at elevated temperatures [9]. The Ru/C catalysts are also highly active in oxidation reactions. For example, according to comprehensive reviews of Stüber et al. [10] and Zhou et al. [11], Ru exhibited the best activity compared to other supported noble metals in catalytic wet air oxidation (CWAO) of organic compounds. However, when ruthenium is supported on carbons, metal leaching and formation of carbonaceous deposits proceed very frequently and therefore a study on the stability of Ru/C catalysts is strongly required.

Currently, a wide range of carbon materials is used for the synthesis of carbon-supported catalysts, i.e., graphite, activated carbon, carbon black, carbon nanotubes, carbon nanofibers, micro/mesoporous carbon networks, graphene, carbon dots, etc. One of the well-known approaches to increasing the adhesion of active components on the carbon surface is doping carbon with nitrogen. The positive role of N-doped carbon nanomaterials (N-CNMs) in the properties of supported Pt-group metals and transition metals (Co, Fe, Ni, Cu) was described in comprehensive thematic reviews [12,13,14]. The use of in situ and ex-situ methods of CNMs doping [14] makes it possible to synthesize various N-CNMs, including the bamboo-like N-doped carbon nanotubes. It should be noted that the first and most representative experimental work on the in situ synthesis of N-CNMs by the use of chemical vapor deposition (CVD) was devoted to the preparation of bamboo-like N-CNTs [15].

Presently, N-CNTs are successfully used for the synthesis of the 4%Ru/N-CNTs catalyst for the hydrogenation of levulinic acid [4]. The catalyst was slightly deactivated in the course of the reaction due to ruthenium agglomeration and the nanoparticle size increased from 1.7 to 2.3 nm. Promising results of using N-CNTs as a Ru catalyst support have been demonstrated in the ammonia decomposition reaction [16,17,18]. The high intrinsic activity of N-CNTs in CWAO of oxalic acid was found [19,20], while according to the results of Rocha et al. [19], N-CNTs are deactivated during the reaction. The authors attributed their deactivation to the fact that CWAO by-products usually induce a partial deactivation of carbons. It should be noted that in all the above works [4,16,17,18,19,20], except for [17], N-CNTs were obtained by the ex-situ method using as-synthesized CNTs. When using this method, N-CNTs retain the morphology of the parent CNTs and specific regular bamboo-like arches are not formed. Thus, the room for the study of bamboo-like N-CNTs stability as a Ru catalyst support in the oxidizing and reducing environment is still open.

In this work, we synthesized bamboo-like N-CNTs by CVD of ethylene-ammonia mixtures on the Fe-Ni catalyst. The synthesized N-CNTs were used as a support for the deposition of ruthenium catalyst. The properties of N-CNTs, 3%Ru/N-CNTs, and their N-free counter partners (CNTs and 3%Ru/CNTs) have been studied in detail by transmission electron microscopy (TEM), high-angle annular dark-field scanning transmission electron microscopy (HAADF-STEM), X-ray photoelectron spectroscopy (XPS), and CO chemisorption methods. Using the CWAO of PhOH and gas-phase FAD as an example, the activity and stability of 3%Ru/N-CNTs catalysts in reactions proceeding in the oxidizing and reducing environment were studied. The CWAO of PhOH belongs to the industrially important processes of wastewater treatment for the removal of organic pollutants [10,11], whereas the FAD is currently considered a promising process for hydrogen production from renewable biomass sources [21,22]. Based on the obtained catalytic data and the results of the study of catalysts after the reactions, conclusions about the stability of bamboo-like N-CNTs as a Ru catalyst support under various conditions are drawn.

## 2. Materials and Methods

### 2.1. Samples Preparation

N-free and N-doped carbon nanotubes were synthesized by the CVD technique. CVD is currently the most common technique to grow both CNTs and N-CNTs. It is known that the formation of carbon nanotubes on metals of the iron subgroup proceeds by the following steps: (1) catalytic decomposition of a hydrocarbon on the surface of a metal particle to form atomic carbon; (2) dissolution of carbon atoms and diffusion through the metal particle; and (3) precipitation at the rear side of the nanoparticles to form a carbon nanofilament [23]. According to the literature, the mechanism of N-CNTs growth is similar to the growth mechanism of undoped CNTs. In addition, the decomposition of the nitrogen-containing precursor produces nitrogen atoms adsorbed on the catalyst surface, which then diffuse, similar to carbon atoms, over the catalyst particle surface or through its bulk [24,25]. In the current work, CNTs were synthesized by CVD of ethylene at 700 °C, and N-doped carbon nanotubes by CVD of ethylene-ammonia mixtures in a flow reactor at 700 °C on a 62%Fe-8%Ni-30%Al_2_O_3_ catalyst [26]. The concentration of ammonia in the ethylene-ammonia mixtures was 25, 40, and 60 vol.%. At the end of the process, the reactor was cooled in an argon flow. The 62%Fe-8%Ni-30%Al_2_O_3_ catalyst was synthesized by a co-precipitation technique using aqueous solutions of metal nitrates (Acros Organics, Waltham, MA, USA) and ammonium bicarbonate (Reachem, Moscow, Russia). The resulting precipitates were first calcined in an air flow at 450 °C for 3 h and then reduced in a hydrogen flow at 580 °C for 5 h. The synthesized CNTs and N-CNTs were washed from the growth catalyst first in concentrated hydrochloric acid at room temperature and then in 2M HCl at 100 °C for 6 h. 

In the current work, we synthesized catalysts (Ru/CNTs and Ru/N-CNTs) with a constant ruthenium content (3 wt.%). The Ru content of 3 wt.% corresponds well to the range of Ru content in usual Ru/C catalysts (typically, within 1–5 wt.%) [10,11]. Ruthenium was deposited on CNTs and N-CNTs by the wetness impregnation technique using an aqueous solution of Ru(NO)(NO_3_)_3_ (Alfa, 31.3 wt.% of Ru) followed by reduction of the samples in a hydrogen flow at 300 °C for 2 h [27]. In the incipient wetness impregnation method, the defined volume of the Ru(NO)(NO_3_)_3_ aqueous solution was added to the freshly dried CNTs or N-CNTs at room temperature, the sample was thoroughly stirred, stored in a closed vessel for 30 min, and then dried in air for 24 h. The volume of the Ru(NO)(NO_3_)_3_ aqueous solution was defined from the water capacity of the carbons. The water capacity of the carbons was measured by the standard incipient wetness impregnation method. Distilled water was dropwise added under stirring to the sample preliminarily dried in Ar at 170 °C until the pores of the material were completely filled. The volume of the absorbed water (cm^3^/g) was determined by weighing. The expected ruthenium content in the catalysts was confirmed by the X-Ray fluorescence spectrometry (Perform’X Spectrometer Thermo Fisher Scientific, Waltham, MA, USA).

### 2.2. Characterization by Physicochemical Methods 

The texture characteristics of the samples were studied by N_2_ adsorption at 77 K using an ASAP 2400 automatic setup (Micrometrics, Norcross, GA, USA). Before the analysis, the samples were evacuated at 150 °C for 24 h.

TEM data were obtained by the use of JEM-2200 FS (JEOL Ltd., Akishima, Japan). The samples were also studied by the use of a double aberration-corrected Thermo Fisher Scientific Themis Z electron microscope. Images with a high atomic number contrast were obtained using a high-angle annular dark field (HAADF) detector in Scanning-TEM (STEM) mode. The spectrum imaging data were recorded using a Super-X G2 EDX detector (Thermo Fisher Scientific). The samples for the TEM study were dispersed ultrasonically and deposited on copper grids covered with a holey carbon film. 

XPS study of the samples was performed on an ES-300 (KRATOS Analytical, Manchester, UK) photoelectron spectrometer with an AlK_α_ source (hν = 1486.6 eV). The spectrometer was calibrated using the Au4f_7/2_ and Cu2p_3/2_ lines of pure metallic surfaces of gold and copper with the corresponding binding energy (E_b_) values 84.0 eV and 932.7 eV. Samples were fixed on the sample holder with carbon scotch tape and analyzed without preliminary pretreatments. The experimental curves were fitted with a combination of Gaussian and Lorentzian peaks after the Shirley background subtraction procedure. The data were processed and analyzed using the XPS-Calc program tested before on carbon-based systems [9,27].

CO chemisorption on the catalysts was measured at room temperature by a standard pulse technique, using He as the carrier gas. Before the measurements, the catalysts were re-reduced in situ in flowing hydrogen (typically, at 150 °C for 15 min in the case of initial samples and 250 °C, 15 min, after using the samples in catalytic reactions), then cooled to room temperature and purged with He. In special cases (to be specified in the text), the samples were subjected to oxidative treatment under a flow of 5%O_2_ in He at 150–200 °C and reduced again under H_2_. The fractions of metal atoms exposed (so called dispersions, Ru_surface_/Ru_total_) were calculated by assuming that the stoichiometry of adsorption CO/Ru_surface_ is equal to 1.

The content of Ru and Fe in the solutions after the CWAO reaction was determined by inductively coupled plasma-atomic emission spectrometry (ICP-AES) using a Perkin-Elmer instrument OPTIMA 4300 (Waltham, MA, USA).

### 2.3. Catalytic Activity Tests

#### 2.3.1. CWAO of PhOH

Catalytic tests were carried out in a high pressure autoclave (Autoclave Engineers, Erie, PA, USA) in an oxidizing atmosphere (20%O_2_/80%N_2_) at a temperature of 160 °C under intense stirring (1500 rpm). The reactor was loaded with 75 mL of a phenol solution with an initial substrate concentration of 21 mmol/L, and 125 mg of the catalyst was added. The autoclave was purged three times with argon and three times with a mixture of 20%O_2_/80%N_2_; the gas pressure was set to 50 atm. The autoclave was then heated to a reaction temperature of 160 °C and the zero probe was analyzed. During the reaction, samples of the reaction solution were taken from the autoclave to analyze the concentration of PhOH and total organic carbon (TOC) after 0.5, 1, 2, 3, 4, 5, and 6 h of the reaction.

The PhOH concentration in the solutions was analyzed by high-pressure liquid chromatography using a MiliChrom A02 chromatograph (Ekonova, Novosibirsk, Russia) equipped with a spectrophotometric detector and a Nucleosil C-18 column thermostated at 35 °C. The eluent (80% 0.05 M ammonium acetate—20 vol.% acetonitrile) was supplied at the 0.15 mL/min flow rate. TOC analysis was performed on an Analytik Jena Multi N/C 2100 S (Jena, Germany).

The PhOH conversion (X_PhOH_, %) and TOC balance were determined using the following formulas:

(1) $$X_{\mathrm{PhOH}}\left(\%\right)=\frac{C_{\mathrm{PhOH}}^{0}-C_{\mathrm{PhOH}}}{C_{\mathrm{PhOH}}^{0}}\;\cdot 100,$$

(2) $$\mathrm{TOC}\left(\%\right)=\frac{C_{\mathrm{TOC}}}{C_{\mathrm{TOC}}^{0}}\;\cdot 100,$$

where $C_{\mathrm{PhOH}}^{0},$ $C_{\mathrm{PhOH}},$ $C_{\mathrm{TOC}}^{0}$, and $C_{\mathrm{TOC}}\;$are the initial and current concentrations of PhOH and organic carbon. 

#### 2.3.2. Gas-Phase FAD 

Gas-phase FAD was carried out in a flow setup using a quartz reactor (internal diameter 6 mm). A 20 mg sample of the catalyst was uniformly mixed with 0.5 cm^3^ of quartz (the 0.25–0.5 mm fraction). Before testing, the catalyst in the reactor was treated with H_2_ (10%H_2_/He) for 1 h at 300 °C. The reactor was then cooled to room temperature and purged with helium. After that, the initial mixture 5 vol.% FA/He preheated to 60 °C was fed into the reactor at a flow rate of 20 cm^3^/min. Catalytic experiments were carried out in a temperature-programmed mode, 2 deg/min. The course of the reaction was monitored by the release of CO and CO_2_, which were analyzed using a gas chromatograph (Tsvet-500, Moscow, Russia). For determination of CO and CO_2_, they were separated on a 1.5 m × 3 mm steel column filled with Porapak-Q at 293 K followed by methanation and analysis with a flame-ionization detector. The FA conversion was determined as a ratio of the sum of CO and CO_2_ concentrations to the initial FA concentration. The selectivity of CO_2_ (H_2_) formation was determined as a ratio of CO_2_ concentration to the sum of CO and CO_2_ concentrations.

The apparent turnover frequency (TOF_app_) of the reaction was calculated as a ratio of the reaction rate obtained at conversions below 20% to the total number of Ru atoms in the catalyst. Additionally, the TOF values were calculated from the number of surface Ru atoms, which was estimated using the TEM size of Ru nanoparticles. The reaction rate (W) was calculated according to the formula:

(3) $$W=\frac{C_{\mathrm{FA}}^{0}\times x\;\times V\;\times N_{A}}{m\;\times V_{m}}\;,$$

where $C_{\mathrm{FA}}^{0}\;$is the initial FA concentration, x—the FA conversion, V—the feed rate, N_A_—Avogadro’s mole number, m—the Ru loading, and V_m_—the molar volume. 

## 3. Results and Discussion

### 3.1. Properties of Carbon Nanotubes and Supported Ru Catalysts

**Figure 1:**
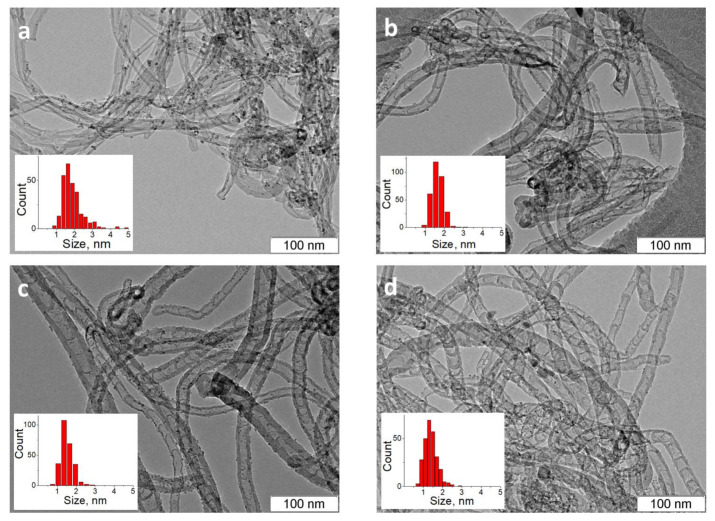
TEM photos of Ru/C catalysts: (**a**) Ru/CNTs, (**b**) Ru/1.9%N-CNTs, (**c**) Ru/3.0%N-CNTs, and (**d**) Ru/4.8%N-CNTs. The Ru nanoparticle size distributions are shown in the insets.

**Figure A1:**
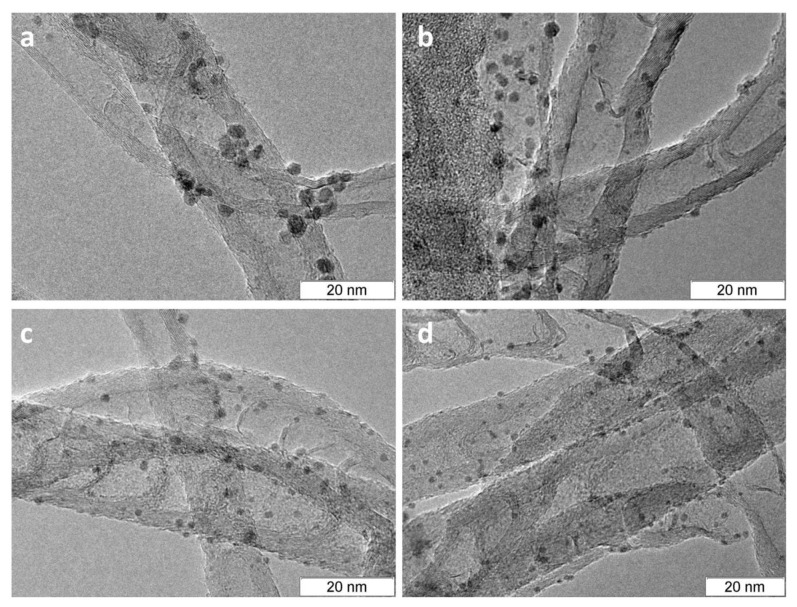
TEM photos of Ru/C catalysts: (**a**) Ru/CNTs, (**b**) Ru/1.9%N-CNTs, (**c**) Ru/3.0%N-CNTs and (**d**) Ru/4.8%N-CNTs.

The TEM study has shown that the decomposition of pure ethylene on the 62%Fe-8%Ni-30%Al_2_O_3_ catalyst leads to the formation of multi-walled carbon nanotubes, Figure 1 and Figure A1. This differs from N-CNTs, where the graphene layers begin to curve, and regular internal arches are formed. It is known that when CVD is used for N-CNTs synthesis, the incorporation of nitrogen into internal and external graphene layers leads to their curvature [28]. The formation of a bamboo-like structure indicates the implementation of a pulsation mode during the growth of N-CNTs [29], in contrast to the continuous growth of CNTs. The specific surface area of the CNTs/N-CNTs varies in the range of 150–169 m^2^/g, and the average pore diameter varies from 11 to 17 nm, Table A1. The obtained textural characteristics are typical of the N-CNTs synthesized by CVD at moderate temperatures [30].

Figure 1 and Figure A1 show representative TEM images of the supported Ru catalysts at low and high magnifications. One can see that all the samples contain rather small Ru particles, but the particle size is dependent on the nitrogen content in the N-CNTs. The largest Ru particles, 2.3 nm, are observed on the N-free CNTs, while the smallest ones, 1.5 nm, are detected on the 4.8%N-CNTs with the maximum content of nitrogen (Table 1). One can also see in Figure 1 and Table 1 that the use of N-CNTs results in the narrowing of the particle size distributions; mean square deviations for N-doped and N-free catalysts differ by a factor of two. These observations are in full accordance with the numerous literature data, which indicate the ability of N-CNMs to stabilize ultrafine nanoparticles and even single atoms [12,13,31,32].

**Figure 2:**
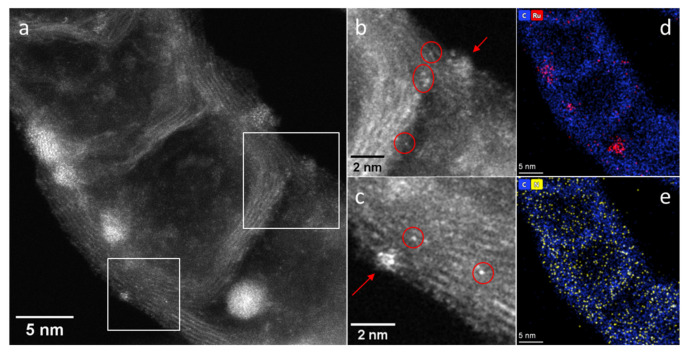
(**a**) HAADF-STEM image of the Ru/4.8%N-CNTs catalyst; (**b**,**c**) enlarged images of the areas marked by squares in Figure (**a**) with brightness/contrast correction for better visualization; the circles indicate the location of single Ru atoms, the arrows indicate the location of Ru clusters; (**d**,**e**) EDX mapping patterns showing the distribution of C (blue), Ru (red) and N (yellow) in the selected region. The maps are presented in background-corrected intensities.

It is also of interest to compare the Ru dispersions derived from TEM with those measured with CO chemisorption. By comparing the corresponding data in the third and fourth columns of Table 1, one can see that the N-doped catalyst Ru/1.9%N-CNTs has an increased ability to chemisorb CO, as compared to the N-free catalyst Ru/CNTs. This correlates with the TEM data, which indicates a higher dispersion for Ru in the former sample. However, the increase in CO/Ru value (0.52/0.45 = 1.15) is much less than predicted by TEM (0.78/0.57 = 1.37). Moreover, TEM predicts an increase in the Ru dispersion from 0.78 to 0.88 upon increasing the N content in CNTs from 1.9 to 4.8%, whereas CO/Ru values for these samples remain the same. This seeming contradiction between the results of the two methods finds an easy explanation if we take into account that nitrogen in N-CNMs should induce positive charging of supported metal species, which limits the back-donation of metal d-electrons to π* antibonding orbital of CO and weakens the metal–CO bond [32]. We have repeatedly seen such an effect in our previous studies with various N-doped catalysts that contained a large proportion of metal clusters and single atoms [33,34,35]. It is known that supported metals interact with nitrogen centers of N-doped carbons and, as a result, the formation of nanoparticles due to coalescence upon heating is prevented [32]. For example, we demonstrated the stability of single Pd-N species in Pd/N-CNTs upon heating in hydrogen up to 500 °C [35]. So, in fact, the CO/Ru values in Table 1 confirm a strong interaction between the N species of N-CNTs and Ru, which results in the formation of positively charged small metal particles and leaves a portion of Ru as single atoms. Indeed, according to HAADF-STEM images, the N-doped catalyst Ru/4.8%N-CNTs contains both the nanoparticles and single atoms, Figure 2. 

**Figure A2:**
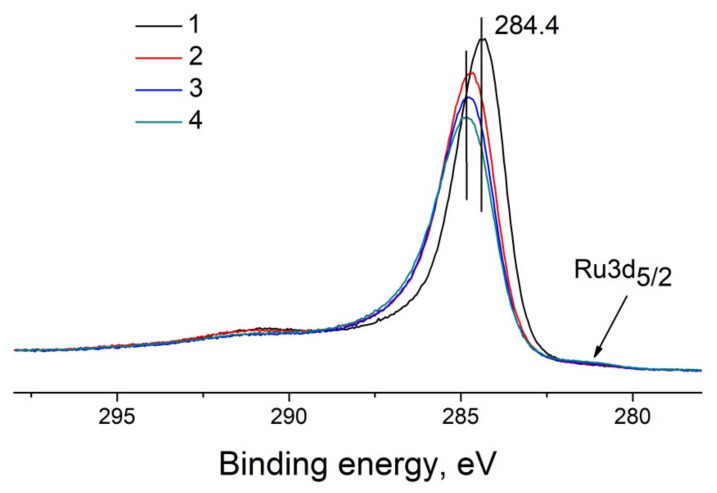
C1s spectra of CNTs (1), 1.9%N-CNTs (2), 3.0%N-CNTs (3) and 4.8%N-CNTs (4).

The C1s spectra of carbons (Figure A2) are characterized by the main intense peak at 284.4–284.8 eV, which is characteristic of sp^2^ carbons [36]. As can be seen, the doping of CNTs with nitrogen is accompanied by a typical shift of the C1s peak towards higher binding energies by 0.4 eV due to the incorporation of nitrogen into the carbon structure [36,37].

**Figure 3:**
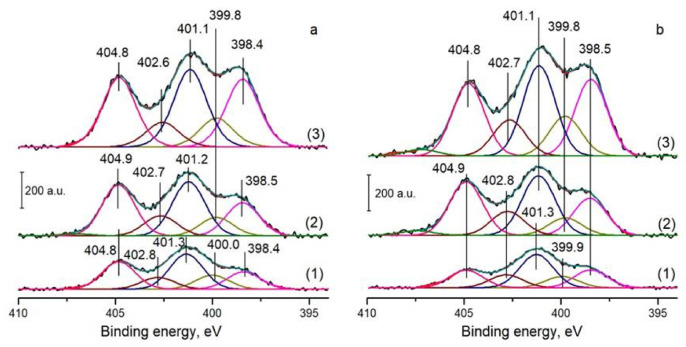
N1s spectra of N-CNTs (**a**) and Ru/N-CNTs (**b**): (1) 1.9%N-CNTs, (2) 3.0%N-CNTs, and (3) 4.8%N-CNTs.

The N1s spectra of N-CNTs contain three main peaks with the binding energies of ~398, ~400, and ~401 eV, which can be assigned to the pyridinic (N_Py_), pyrrolic (N_Pyr_), and graphitic (N_Q_) nitrogen species, respectively [25,38], Figure 3a. The spectra also show an intense peak at 402.6–402.8 eV, which corresponds to an oxidized state of nitrogen. The peak at 404.8–404.9 eV can be attributed to molecular nitrogen encapsulated inside bamboo-like N-CNTs [25]. As can be seen from the data presented, the predominant nitrogen species in N-CNTs is graphitic nitrogen, but an increase in the nitrogen content is accompanied by a monotonic increase in the N_Py_/N_Q_ ratio from 0.5 to 0.9, Table A2. 

**Figure 4:**
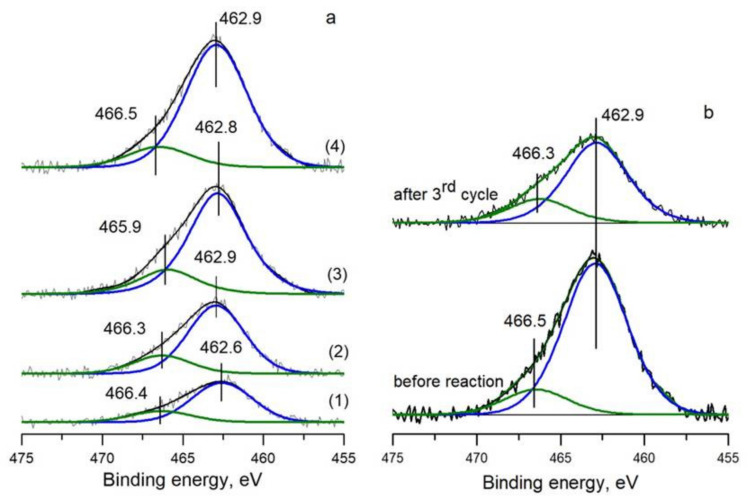
(**a**) Ru3p_3/2_ spectra of Ru/CNTs (1), Ru/1.9%N-CNTs (2), Ru/3.0%N-CNTs (3) and Ru/4.8%N-CNTs (4); (**b**) Ru3p_3/2_ spectrum of 3%Ru/4.8%N-CNTs after CWAO of PhOH.

The Ru3p_3/2_ spectrum contains the main maximum at 462.6–462.9 eV and a weak maximum at 465.9–466.5 eV, Figure 4. Based on the literature data on the binding energy of metallic Ru^0^ (461–462 eV) and oxidized Ru^4+^ (463–464 eV), it can be assumed that rather small nanoparticles of ruthenium are present in the catalysts [39,40,41]. The shift of the main peak for N-doped catalysts toward higher binding energies by 0.2–0.3 eV may be due to both the decrease in the size of nanoparticles [42] and the interaction of the metal with pyridinic nitrogen centers of N-CNTs (Ru^δ+^–N_Py_) [38,43,44]. The peak at ~466 eV can be attributed to ruthenium hydroxides with the general formula RuO_x_H_y_ [41]. It is interesting to note that close values of the binding energy of ruthenium (E_b_(Ru3p_3/2_) = 462.6 eV) were observed when ruthenium was deposited on N-CNFs (herring-bone carbon nanofibers), which were synthesized by a similar CVD method, and the size of ruthenium nanoparticles was also 1.5 nm [27]. It should be noted that Meng et al. [4] reported the formation of electron-rich Ru species in the 4%Ru/N-CNTs catalyst with a ruthenium nanoparticle size of 1.7 nm; the N-CNTs were synthesized by post-treatment of parent CNTs by pyridine. It can be supposed that different methods of N-CNT synthesis lead to the formation of external graphene layers with different ratios of main nitrogen species (N_Q_ and N_Py_); as a result, ruthenium can be stabilized at different nitrogen centers. For example, we have shown that N-CNTs synthesized by CVD and post-treatment of oxidized CNTs with ammonia exhibit different intrinsic activities in the aerobic oxidation of syringyl alcohol due to significant differences in the ratio of graphitic and pyridinic nitrogen in the tubes [26].

The analysis of the N1s spectra of N-doped catalysts shows no significant changes compared to the spectra of N-CNTs, Figure 3b. At present, it is not possible to make a conclusion about the interaction of a certain nitrogen species with supported ruthenium. To increase the sensitivity of the XPS analysis, it is preferable to carry out this study using low photon energy [43]. However, taking into account the data showing the poor ability of the N-doped catalysts to CO chemisorption, it can be supposed that some part of Ru in the catalysts interacts with pyridinic nitrogen (Ru^δ+^−N_Py_) [38,43].

### 3.2. Activity and Stability of the Carbons and Ru/C Catalysts in Oxidizing Environment: CWAO of PhOH

**Figure 5:**
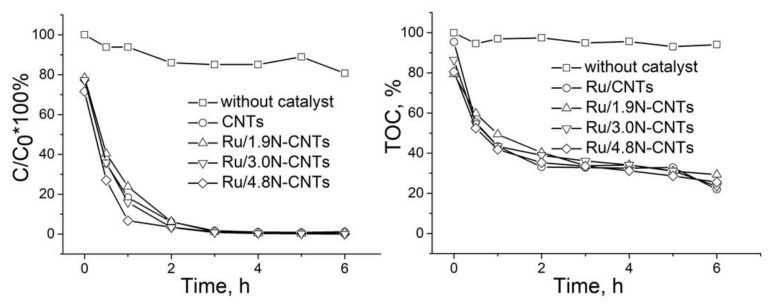
Kinetic curves of PhOH oxidation on Ru/C catalysts.

**Figure A3:**
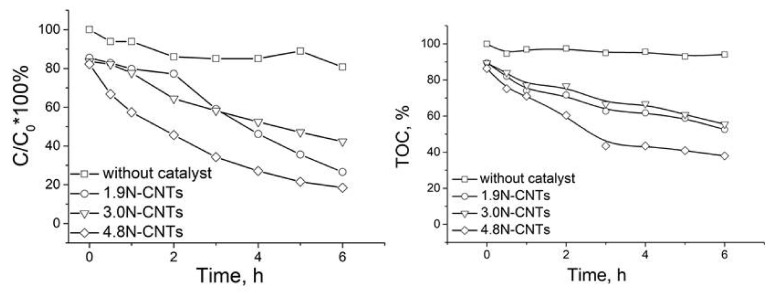
Kinetic curves of PhOH oxidation on N-CNTs.

**Figure A4:**
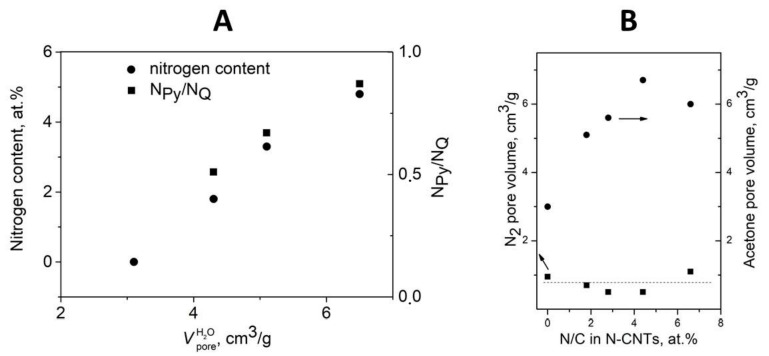
(**A**) Effects of nitrogen content and N_Py_/N_Q_ ratio on water capacity of N-CNTs; (**B**) N_2_ pore volume and acetone capacity of the CNTs and N-CNTs. (**A**) Reprinted from [53], Copyright 2018, with permission of American Chemical Society. (**B**) Reprinted from [54], Copyright 2021 by the authors.

In Figure 5, data on non-catalytic WAO and CWAO are presented. Without a catalyst, PhOH oxidizes slowly; after 6 h of reaction, the conversion does not exceed 15%. As can be seen from Figure A3, N-CNTs exhibit intrinsic activity, PhOH conversion reaches ca. 60–80% after 6 h of the reaction, while according to TOC data, ca. 40–60% of the initial organic carbon remains in solution and is not oxidized to CO_2_ and H_2_O. It is known that carbon nanomaterials are good adsorbents of organic compounds and also exhibit activity in CWAO [10,11,45]. It is interesting to note that a decrease in the phenol concentration in the presence of carbon nanotubes is observed at the “zero” point, which may indicate the adsorption of PhOH on carbons before the reaction begins: the PhOH concentration at the zero point decreases by 15–18% in the presence of N-CNTs, Figure A3. Indeed, the high affinity of N-CNTs to water and organic solvents is known, as seen in Figure A4. The highest activity was demonstrated by N-CNTs with the highest nitrogen content, 4.8% N-CNTs. It is reported that the basic pyridinic nitrogen species of N-CNMs favor CWAO reactions [19,20]. As can be seen from Table A2, an increase in the nitrogen content in N-CNTs is accompanied by an increase in the content of pyridinic nitrogen and, as a result, the maximum content of N_Py_ is recorded in 4.8% of N-CNTs.

The deposition of ruthenium on carbons is accompanied by a significant acceleration of the reaction rate, the PhOH conversion reaches 94–97% after 3 h of the reaction and PhOH is totally decomposed after 3 h of the reaction on all the catalysts, Figure 5. The analysis of the TOC values shows that the supported ruthenium promotes the deep oxidation reaction compared to CNTs/N-CNTs, and the TOC value decreases to ca. 20–30%, Figure 5. According to TEM, the size of ruthenium nanoparticles in the supported catalysts varies in a narrow interval (1.5–2.3 nm) and, therefore, their catalytic properties seem to be similar. In view of that, it can be assumed that the atomic Ru species in N-doped catalysts do not accelerate the CWAO of PhOH. Indeed, Gallezot et al. reported for Ru/C catalyst that when the size of ruthenium decreases from 2 nm to less than 1 nm, the reaction rate of acetic acid oxidation decreases by a factor of 9 [46]. The authors explained this effect by the higher adsorption energy of oxygen on the sub nano particles. A similar increase in the rate of the CWAO of acetic acid with an increase in the size of ruthenium nanoparticles deposited on Ni-NCNTs was also observed by Jin et al. [47]. 

**Figure 6:**
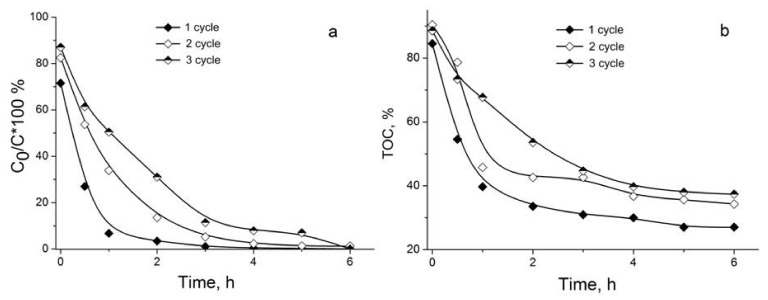
Cycling of Ru/4.8%N-CNTs in CWAO of PhOH: time dependences of PhOH conversion (**a**) and TOC (**b**).

The catalyst Ru/4.8%N-CNTs with the highest degree of N doping was investigated in cycling experiments. It turned out that the catalyst is not stable in the reaction; although 100% conversions were still achievable in repeated runs, the reaction was slowed down sequentially in the second and third cycles. After 1 h of the reaction, the conversion of PhOH in the first cycle reached ca. 95%, in the second one decreased to ca. 60%, and the third did not exceed 50%, Figure 6a. The catalyst deactivation is confirmed by a 10% increase in the TOC value in the second and third cycles, Figure 6b.

It should be noted that the deactivation of carbons in CWAO is discussed in the literature [10,45]; however, positive examples of the use of these materials are also described. For example, chemically modified CNTs showed stability in the CWAO of phenol [48]; N-CNTs synthesized by ball milling of pristine CNTs with melamine or urea were only slightly deactivated during three consecutive runs [20]. To elucidate the reasons for catalyst deactivation when using bamboo-like N-CNTs, the sample after the reaction was studied by XPS, TEM, and CO chemisorption.

**Figure A5:**
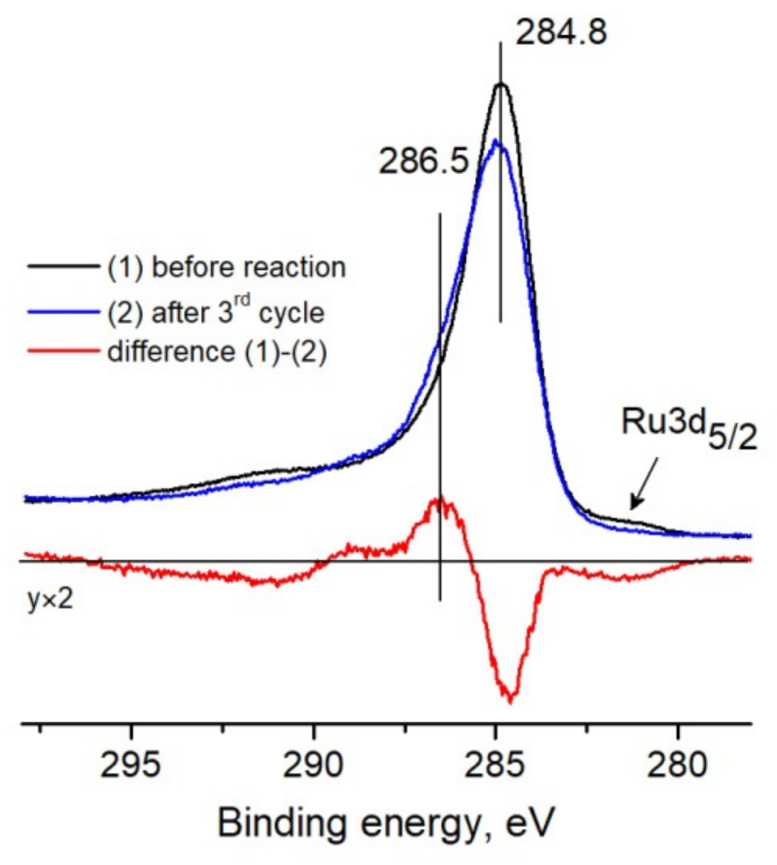
C1s spectra of Ru/4.8%N-CNTs catalyst before and after CWAO of PhOH.

According to XPS, the state of ruthenium in the catalyst after three consecutive runs remains stable, and the binding energy of ruthenium E_b_(Ru3p_3/2_) does not change, 462.9 vs. 462.9 eV, Figure 4b. At the same time, the Ru/C ratio decreases from 0.7 to 0.4, which suggests that the ruthenium surface is shielded by reaction by-products or that ruthenium is sintered during the reaction. In turn, in the C1s spectra (Figure A5), an increase in intensity is observed in the region of 286 eV, which may be associated with the formation of alcohol and/or carbonyl (E_b_ (C1s) ~286–287 eV) oxygen-containing groups on the carbon surface [49]. We also recorded a decrease in the signal intensity at ca. 284.5 eV, which may be due to the formation of amorphous carbon on the support surface and/or the oxidative destruction of N-CNTs. The latter is confirmed by an increase in the XPS O_at_/C_at_ ratio (%) from 4.5 to 15 after the first cycle and to 29 after the third cycle.

**Figure 7:**
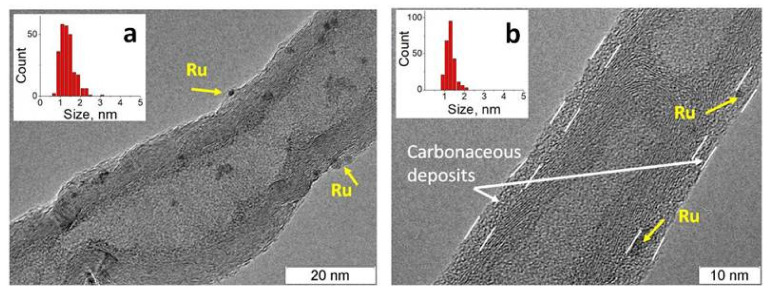
TEM photos of the catalyst Ru/4.8%N-CNTs after the first (**a**) and third (**b**) cycles of CWAO of PhOH.

According to TEM, after the first cycle, the mean size of ruthenium nanoparticles does not change, 1.5 nm vs. 1.6 nm, as seen in Figure 7. It can be seen that some nanoparticles are encapsulated in a carbon shell. After the thirrd cycle, extended carbonaceous deposits are formed on the surface of the tubes, which, most likely, block ruthenium nanoparticles. It should be noted that the mean size of ruthenium nanoparticles also does not change after the third cycle.

**Figure 8:**
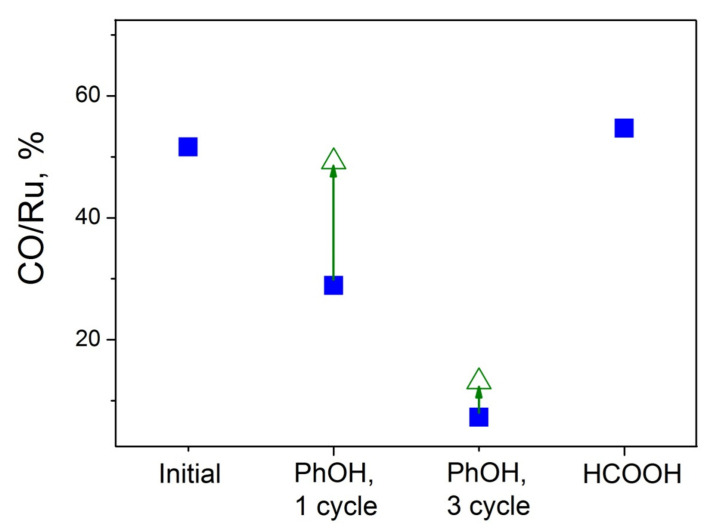
Results of CO chemisorption measurements for the Ru/4.8%N-CNTs catalyst before and after catalytic testing in CWAO of PhOH and long-term FAD. Open symbols (triangles) refer to the samples after oxidative cleaning (see details in the text).

Summarizing the TEM and XPS data, we can state that catalytic testing of the samples in CWAO of PhOH leaves the size of Ru nanoparticles constant but provokes coverage of the Ru surface with carbonaceous species, with the extent of such coverage being increased with time. This finds is further confirmed by the CO chemisorption results in Figure 8. Here, blue squares correspond to the CO adsorption ability of the samples after standard pretreatment before the measurements (re-reduction of air-contacted samples, as described in Section 2.2). By considering these data, one can see that CO/Ru values for the samples after catalysis are strongly decreased, especially if the sample was repeatedly used in three catalytic cycles. This is in line with expectations for metal particles whose surface is partially blocked by carbonaceous deposits. It is also of interest to compare CO/Ru values for the same catalysts after additional oxidative treatment. Following the first measurements (blue symbols in Figure 8), the samples were treated in situ in a flow of 5%O_2_/He at 200 °C for 5 min, then re-reduced, and CO chemisorption was repeated; these new data are shown in Figure 8 by green triangles. It is clearly seen that such a treatment restores the adsorption ability of the first sample (after one catalytic cycle) to the level characteristic of this sample before catalysis. However, the oxidative cleaning under these conditions turns out ineffective if three catalytic runs were performed. The latter indicates the formation of a thicker and/or denser and, so, less reactive carbon layer upon increasing the duration of catalytic testing in the given reaction.

It should be noted that the analysis of the solution after the reaction showed the presence of a significant amount of iron (3 wt.%). It seems that iron leaching proceeds as a result of the partial oxidation/destruction of N-CNTs in the course of the reaction. A minor loss of ruthenium after the reaction (0.05 wt.%) was also observed, thus proving the instability of the catalyst. Therefore, it can be concluded that bamboo-like N-CNTs synthesized by CVD are unstable in the oxidative environment at elevated temperatures. The curved N-containing graphene layers, forming a specific bamboo-like structure, are more easily oxidized compared to nitrogen-containing graphene layers in which nitrogen is embedded by the substitution of O-containing groups [20,48].

### 3.3. Activity and Stability of the Carbons and Ru/C Catalysts in Reducing Environment: Gas-Phase FAD

**Figure 9:**
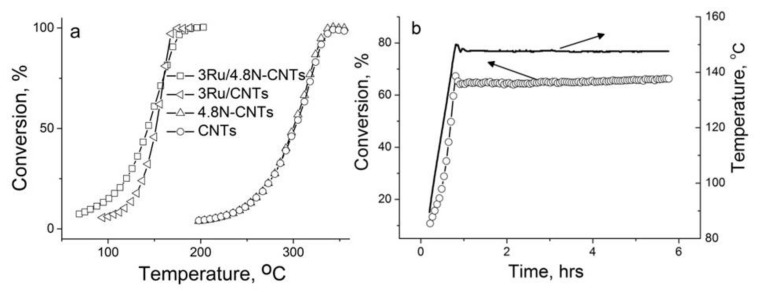
Temperature dependences of FA conversion on the carbons and Ru/C catalysts (**a**); and stability test of the Ru/4.8%N-CNTs in the course of the reaction (**b**).

The Ru/4.8%N-CNTs catalyst with the highest degree of N doping and N-free Ru/CNTs counter partner for comparison were studied in the gas-phase FAD. Earlier we reported a monotonic increase in the reaction rate with the degree of nitrogen doping for 1%Pt/N-CNFs [50]. At the beginning we tested the intrinsic activity of the carbons; both the N-doped and N-free CNTs showed activity at temperatures higher than 200 °C, Figure 9a. This result is in good agreement with the results on low activity in this reaction of other N-CNMs, for example, herring-bone N-CNFs [50] or N-doped porous carbon [51]. The deposition of ruthenium on carbons shifts the temperature of 50% FA conversion to low temperatures by ca. 150 °C, with the most pronounced effect being observed for Ru/4.8%N-CNTs. The apparent TOF_100C_ values for Ru/4.8%N-CNTs and Ru/CNTs were found to be 0.019 and 0.008 s^−1^, respectively. A comparison of TOF values calculated per the number of surface atoms (0.022 vs. 0.014 s^−1^) shows that the observed differences cannot be explained by a decrease in the size of ruthenium nanoparticles. In addition, the use of N-CNTs resulted in an increase of the selectivity to hydrogen by 13%, which corresponds to a 2-fold decrease in the CO concentration. Apparently, the presence of sub-nanoparticles and single atoms in the Ru/4.8%N-CNTs makes a positive contribution to the rate and selectivity of the reaction, similarly to [33,35,43]. Indeed, Pd, Pt, Au, Co, Ni, and Cu in the form of single atoms show high activity and selectivity in the FA decomposition reaction toward hydrogen production [32].

It is interesting to note that the apparent TOF_100C_ value for 3%Ru/4.8%N-CNTs (0.019 s^−1^) is very close to the TOF_100C_ value for 1%Ru/6.8%N-CNFs (0.016 s^−1^) [9]. The latter catalyst also contained nanoparticles ca. 1.5 nm in size and single atoms. Therefore, we can conclude that ruthenium interacts with N-CNMs of various morphologies (bamboo-like carbon nanotubes or herring-bone carbon nanofibers) in the same way, which results in the formation of nanoparticles of a similar size and single atoms. The lower activity of the N-free catalyst 2%Ru/carbon Norit (TOF_100C_ 0.013 s^−1^) with a ruthenium dispersion of 5.6% [52] confirms the positive role of the nitrogen centers of N-CNTs in the stabilization of ruthenium in the highly dispersed state, which exhibits higher activity in this reaction.

**Figure A6:**
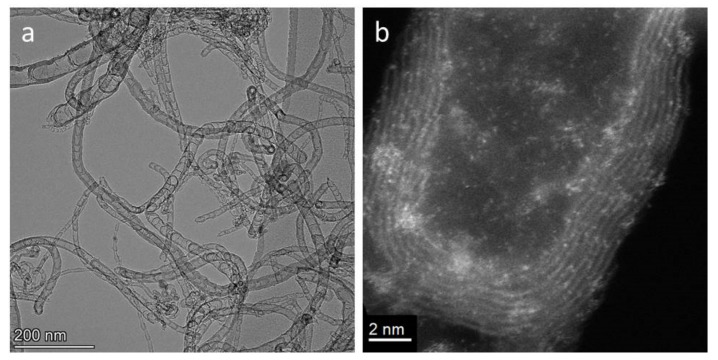
(**a**) TEM and (**b**) HAADF-STEM photos of Ru/4.8%N-CNTs after gas-phase FAD reaction.

The stabilization of ruthenium by N-CNTs and its accessibility is confirmed by long-term experiments. As can be seen from Figure 9b, the catalyst shows stable operation during 6 h of the reaction. Contrasting to the case of phenol oxidation (Figure 8), no decrease in CO/Ru values was observed even after long-term testing of the samples under the conditions of FAD at an elevated temperature of ca. 150 °C. TEM evidently shows the stability of the N-CNTs in the course of the reaction (Figure A6a), while HAADF-STEM confirms the presence of single atoms in the catalyst after the reaction (Figure A6b). Thus, under the conditions of the gas-phase FAD proceeding in a reducing medium at elevated temperatures, the Ru supported on bamboo-like N-CNTs demonstrates high stability concerning its dispersity and accessibility to reagents.

## 4. Conclusions

Bamboo-like N-CNTs have been successfully used as supports for the synthesis of highly dispersed ruthenium catalysts. The deposition of ruthenium on N-CNTs makes it possible to decrease the size of nanoparticles compared to N-free CNTs from 2.3 to 1.5 nm and, in addition, to stabilize ruthenium in the atomic state. The catalysts have been investigated in CWAO of PhOH and gas-phase FAD reactions proceeding at elevated temperatures in an oxidizing and reducing environment.

All 3% Ru catalysts, irrespective of the nitrogen doping, efficiently oxidize phenol at 160 °C, ~100% conversion is achieved after ca. 3 h of the reaction, and TOC values are ca. 30%. On the contrary, a pronounced positive effect of the N-CNTs was observed in the gas-phase FAD, the TOF values for N-free and N-doped catalysts differed by a factor of more than two, and the CO concentration also decreased twofold. The result obtained can be explained by the high sensitivity of the FAD to the presence of single atoms in the catalyst, compared to the CWAO reaction.

Bamboo-like N-CNTs showed different stability in the studied reactions. In the course of CWAO, the catalyst Ru/N-CNTs is deactivated due to partial oxidation of the tubes resulting in the formation of carbonaceous deposits, which block ruthenium nanoparticles. However, the catalyst still shows a high efficiency under three consecutive runs, with 100% PhOH conversion being achieved. During the FAD, the Ru/N-CNTs catalyst exhibits high stability concerning formic acid conversion as a result of the stability of the N-CNTs under these conditions and the accessibility of ruthenium.

## Figures and Tables

**Table 1:** TEM and CO chemisorption data for Ru/C catalysts.

Catalyst	Mean Particle Size by TEM, nm	Dispersion by TEM	Dispersion by Chemisorption (CO/Ru_total_)
Ru/CNTs	2.3 ± 0.6	0.57	0.45
Ru/1.9%N-CNTs	1.7 ± 0.3	0.78	0.52
Ru/3.0%N-CNTs	1.6 ± 0.3	0.83	0.51
Ru/4.8%N-CNTs	1.5 ± 0.3	0.88	0.51

## Data Availability

The data presented in this study are available on request from the corresponding author.

## Appendix A

**Table A1:** Texture characteristics of the carbons.

Sample	S_BET_, m^2^/g	V_Σ_, cm^3^/g	V_micro_, cm^3^/g	Pore Diameter, nm
CNTs	166	0.70	0	17
1.9%N-CNTs	169	0.46	0	11
3.0%N-CNTs	160	0.51	0	13
4.8%N-CNTs	150	0.47	0	13

**Table A2:** XPS data on the nitrogen content and contribution of different nitrogen species in N-CNTs: N_Py_—pyridinic nitrogen (BE N1s~398 eV), N_Pyr_—pyrrolic nitrogen (BE N1s~400 eV), N_Q_—graphitic nitrogen (BE N1s ~ 401 eV), N_Ox_—oxidized nitrogen (BE N1s~402 eV), and N_N2_—encapsulated molecular N_2_ (BE N1s ~ 405 eV).

Support	N_Total_, at.%	N_Py,_ %	N_Pyr_, %	N_Q_, %	N_NOx_, %	N_N2_, %	N_Py_/N_Q_
1.9%N-CNTs	1.9	16.4	13.3	33.3	11.1	25.9	0.5
3.0%N-CNTs	3.0	18.5	10.3	30.3	11.2	29.7	0.6
4.8%N-CNTs	4.8	25.3	10.8	28.8	9.2	25.9	0.9
